# High Prevalences of Current Mental Disorder Diagnoses Among University Students in Sweden: A Cross‐Sectional Study With Implications for Student Mental Health Services

**DOI:** 10.1002/mpr.70080

**Published:** 2026-05-27

**Authors:** Anne H. Berman, Claes Andersson, Marcus Bendtsen, Petra Lindfors, Nooshin Talebizadeh, Naira Topooco, Olof Molander

**Affiliations:** ^1^ Department of Psychology Uppsala University Uppsala Sweden; ^2^ Karolinska Institutet & Stockholm Region Health Services Stockholm Sweden; ^3^ Health and Society Malmö University Malmö Sweden; ^4^ Department of Health Medicine and Caring Sciences Linköping University Linköping Sweden; ^5^ Department of Psychology Stockholm University Stockholm Sweden

**Keywords:** diagnoses, DSM‐5, mental disorders, prevention, treatment, university students

## Abstract

**Objectives:**

To establish diagnostic prevalences for common mental disorders among students at higher education institutions (HEI's) in Sweden.

**Methods:**

This was a cross‐sectional study assessing diagnostic prevalences for 17,948 students at 30‐day, 12‐month and lifetime timepoints, according to the Diagnostic and Statistical Manual, fifth edition (DSM‐5). Sample participants at 15 HEI's answered an online survey 2020‐2023 within the World Mental Health International College Student (WMH‐ICS) initiative. Diagnoses are reported for Major Depressive Disorder (MDD), Generalized Anxiety Disorder (GAD), Panic Disorder (PD), Bipolar Disorder (BD, any), Alcohol Use Disorder (AUD) and Substance Use Disorder (SUD). Multilevel logistic regression was used to estimate odds ratios in relation to diagnoses of any mental disorder.

**Results:**

Respondents (69.4% women, 25.5 mean age, range 18 to ≥ 36) reported 25.2%, 39.3% and 44.5% prevalences for any mental disorder at 30‐day, 12‐month and lifetime periods. Odds for any mental disorder were markedly higher for non‐binary students and those with non‐heterosexual orientation; odds were also higher for students aged 20–29 years, women, heterosexual individuals with same‐sex attraction and post‐pandemic respondents.

**Conclusions:**

The findings augment existing concern over student mental health. Student mental health services should expand menus for low‐threshold treatment interventions, to promote flourishing and resilience.

## 1 Introduction

Globally, just under one billion people are estimated to be suffering from mental disorders. The proportion of all‐cause years lived with disability (YLDs) that can be attributed to mental disorders is close to 16%, and such disorders are now the leading causes of YLDs (WHO 2022). The numbers cited concern pre‐pandemic estimates from 2019. Later figures from the initial phase of the COVID‐19 pandemic show that the prevalence of major depressive disorder jumped from 2471 to 3153 cases per 100,000, with an increase for anxiety disorders from 3825 to 4802 cases per 100,000 (Santomauro et al. 2021). The high prevalence of mental disorders, their significant contribution to YLDs, and the increase in treatment needs seen during the COVID‐19 pandemic all highlight the urgency of reducing the treatment gap (Alonso et al. 2018; Kazdin 2017).

For young people 15–29 years of age, the YLD figures attributable to mental disorder are even higher than for adults overall, reaching to over 25% for men and over 23% for women, in comparison to nearly 16% overall (WHO 2022). Among young adults, disorder prevalence increased more for younger people than for older age groups during the COVID‐19 pandemic, increasing health inequities for younger people (Santomauro et al. 2021). For this age group, grappling with issues of personal identity and life choices characteristic of emerging adulthood, the mental health challenges may be the greatest over the life course (Arnett et al. 2014). In terms of daily occupations, young adults may be divided into two groups—those who opt to enter the job market, and those who opt for higher education. In a comparison of young adults in and out of college around the turn of the millennium, almost half in both groups were found to fulfill criteria for at least one psychiatric disorder, but treatment rates were low in both groups, and lower for alcohol use disorders among the college students (Blanco et al. 2008). About 75% of students have shown reluctance to seek treatment, due to wanting to resolve their problems on their own, a preference for sharing their problems with friends and family, and stigma (Ebert, Buntrock, et al. 2019). A recent study showed that non‐students’ barriers to treatment‐seeking were more likely to be cost‐related than those for students (Cadigan et al. 2019).

Increasing the attractiveness and accessibility of mental health treatment options for both students and young people in the job market is vital to improving their chances of fulfilling their life opportunities. A necessary prior first step is to systematically identify the prevalence of mental disorders among them. Several research initiatives focusing on students have been established for this purpose, including the American College Health Association‐National College Health Assessment (ACHA‐NCHA) initiative (Lederer and Hoban 2022), the US‐based Healthy Minds study (Lipson et al. 2022), and the Norwegian Students' Health and Wellbeing Study (SHOT) (Sivertsen et al. 2023). The globally oriented World Mental Health International College Student (WMH‐ICS) initiative focuses on university and college students and includes 18 partner countries engaged in yearly screening and follow‐up surveys to investigate the onset and persistence of common mental disorders. As part of this initiative, the WMH‐ICS initiative also develops and evaluates digital mental health treatment interventions for the disorders indicating the highest need, tailoring intervention content and tone to the regional culture (Cuijpers et al. 2019). The WMH‐ICS survey focuses on self‐rated common mental disorder symptoms that generate diagnoses based on the fifth edition of the Diagnostic and Statistical Manual for Mental Disorders (DSM‐5; American Psychiatric Association 2013); prevalences of mental disorders among 13,984 students in eight countries have been reported (Auerbach et al. 2018).

Current mental health trends among young people show high prevalences in the most recently reported WHO data (WHO 2022), and demonstrate even higher levels of mental disorders in the student group (Auerbach et al. 2018). Swedish participation in the WMH‐ICS initiative has involved introducing systematic, routine collection of data on mental disorders for the first time among students in Sweden; it also includes evaluation of transdiagnostic internet‐based cognitive‐behavioral therapy (ICBT) for this group (Berman et al. 2024). The purpose of this article is to report past‐month, past‐year and lifetime diagnostic prevalences for mental disorders among students in eight cross‐sectional cohorts from 15 Swedish institutions of higher education, collected between 2020 and 2023. We also estimate associations between student characteristics and odds of diagnosis, including an assessment of any changes in levels of mental disorders over time in relation to course of the COVID‐19 pandemic. From this regional lens, the findings generate implications for treatment development, evaluation, and implementation.

## 2 Methods

Data were collected by inviting university students at 15 institutions of higher education in Sweden to respond to an online cross‐sectional survey on mental health, in eight cross‐sectional cohorts between 2020 and 2023. Seven of the eight cohorts targeted only first‐term students in educational programs. The first cohort diverged in inclusion criteria and sampled two universities, not restricting inclusion to first‐term students.

### 2.1 Participants

The criteria for survey participation included being a first‐term student registered in an educational program leading to a professional degree (e.g., law, medicine, economics, engineering, disciplines in the social sciences and humanities, etc.). A total of 179,976 students were deemed eligible by their institutions to participate in the survey. Following removal of duplicates, 176,605 were invited to participate, of whom 24,030 (13%) consented. Of these, 13,239 (55.1%) completed the entire survey, and an additional 4709 (19.6%) completed at least the first two sub‐sections of the survey, providing sufficient baseline data for imputation of remaining missing variables for calculation of diagnostic algorithms. The remaining 6082 (25.3%) completed too little of the survey for imputation and diagnostic evaluation. A total of 17,948 students (74.7% of consenters) were thus included in this study.

### 2.2 Measures

The survey began with study information including an informed consent query, followed by 11 survey sub‐sections used by the WMH‐ICS consortium, and ending with questions unique to the Swedish WMH‐ICS surveys, not reported here. The WMH‐ICS survey is largely based on the Composite International Diagnostic Interview Screening Scales (CIDI‐SC) (Kessler and Üstün 2004), and includes items with multiple response alternatives in the areas of demographic information, health, attention and concentration, emotional problems, alcohol and drugs, self‐harm including suicidal ideation and behaviors, experiences of seeking treatment, childhood experiences, recent experiences including everyday stress and severe stress, sexuality and self‐esteem. Ethnicity was not specifically queried, but proxy data were collected in the form of participants' and parental country of birth, as is customary in Sweden. The analyses reported here concern diagnostic outcomes, generated from WMH‐ICS DSM‐5 diagnostic algorithms, at Past 30‐day, Past 12‐month and Lifetime periods for Major Depressive Disorder (MDD), Generalized Anxiety Disorder (GAD), Panic Disorder (PD), Bipolar Disorder (BD, any), Alcohol Use Disorder (excepting Lifetime), Substance Use Disorder, and Any mental disorder. All variables of interest were measured in each of the eight cross‐sectional cohorts. Diagnostic outcomes were calculated based on fulfilment of diagnostic criteria reported in responses to survey items. These criteria were coded in R according to algorithms formulated by the WMH‐ICS consortium. The only exception was calculation of Alcohol Use Disorder (AUD) diagnoses, where we adopted the algorithm used in Auerbach et al. (2018), defining AUD as either a total score of at least eight or a score of four on the Alcohol Use Disorder Identification Test (AUDIT) dependence questions (items 4, 5 and 6) (Babor et al. 2001), corresponding to mild and moderate to severe AUD according to the DSM‐5 (American Psychiatric Association 2013).

### 2.3 Procedures

Student contact information including name and e‐mail address, as well as students' personal identity number, area of study, faculty, department and educational program, was shared by each participating university's unit for the national educational registry system. The survey was administered via the Qualtrics platform, from which all e‐mail invitations and up to three automated reminders were sent. At the start of the survey, students were provided with detailed information on the study including contact information to the Principal Investigator (PI), as well as a dedicated e‐mail address for the study, monitored on weekdays; all study information was available for immediate downloading. Directly following the study information, participants were asked to provide their specific consent to survey participation and, separately, to indicate whether they agreed to linking of survey data with educational registry outcomes (the Swedish Ladok system). Only participants who provided their consent to survey participation received access to the survey (regardless of consent to registry linkage). At the end of the survey, participants were provided with a downloadable pdf‐file referring them, as needed, to national healthcare resources as well as to student mental health services at each participating university. The study was approved by the national Swedish Ethical Review Authority; see specific permission dates and numbers in ethics statement at the end of this article.

### 2.4 Statistical Analysis

Primary analyses were conducted using multilevel logistic regression with adaptive intercepts for cohort and university. Prevalences were estimated using models without other covariates and asssociations were estimated by models including each student characteristic separately. Sexual orientation prevalences were reported according to the categories indicated in Auerbach et al. (2018) to facilitate direct comparison; that is, identification as heterosexual or LBGTQ+, with or without same‐sex attraction for the heterosexual group, and with or without same‐sex activity for the non‐heterosexual group.

When estimating associations, reference categories were selected based on one of three criteria: (1) reference groups used in the analysis of WHO‐WMH‐ICS diagnostic categories in eight countries (Auerbach et al. 2018); (2) selecting the group with the most participants, for example for “work in parallel” and “hours of study per week” variables; and (3) using the earliest timepoint for the “Covid‐19 periods” variable, constructed to represent three pandemic phases: early (spring and fall 2020), persisting (spring and fall 2021, and spring 2022), and post‐pandemic (fall 2022, and spring and fall 2023). Missing data were imputed using multiple imputation with chained equations (200 datasets generated using 30 iterations each). See Supporting Information S1 for details of the imputation procedure, as well as Table S1 for a sensitivity analysis comparing diagnostic prevalences in the total imputed sample with those in the partially imputed sample as well as in an analysis of non‐imputed data.

Models were estimated using both maximum likelihood estimation and Bayesian inference. The former created a basis for null hypothesis testing with significance levels adjusted using Bonferroni correction. The latter created a basis for estimating the probability of any association and without dichotomization. In the Bayesian analyses, main intercepts and coefficients for covariates were given (half) Student's t priors (centered at 0, with 3 degrees of freedom and a scale of 2.5). Adaptive intercepts were given normal priors centered at 0 with a standard normal hyperprior for the standard deviation parameter. The medians of the posterior distributions were used as point estimates, accompanied by 95% compatibility intervals (CoI) defined by the 2.5% and 97.5% percentiles of the posterior distributions. We also reported the probability of an association as the proportion of the posterior distribution in the direction of the mean. Further details are available in the pre‐registered analysis plan at https://doi.org/10.17605/OSF.IO/3EQ7Y. R Studio (version 2024.09.0 + 375) was used for maximum likelihood estimation with the lme4 package, and Stan version 2.30.1 was used for Bayesian inference. The STROBE statement checklist protocol for this article is available in Supporting Information S2.

## 3 Results

Frequency‐based and Bayesian inference analyses showed similar patterns overall, both regarding calculations of diagnostic prevalences (Tables 1 and 2) and regarding associations between participant characteristics and diagnoses, in terms of odds ratios (Tables 3 and 4). Sample prevalences are shown for each participant characteristic sub‐group in Table 3. Participants' mean age was 25⋅5 years (SD 7⋅9), with one out of six in the 18–19‐year age group, two‐thirds in the 20–29 years group and one out of five 30 years or older. Women were the majority, men the minority, and 2% identified as “other” genders. Most students had never been married or cohabited with a partner; still, over one in four were married or cohabiting, and one in 20 was separated, divorced or widowed. Most students came from universities sited in metropolitan areas. Most students were born in Sweden, and two‐thirds reported that both parents were born in Sweden, one in 10 reported one parent born in Sweden, while two out of 10 reported both parents as born abroad. One in 20 identified as an international student, defined as a citizen of another country who came to Sweden for their education. Parental education was reported as high (university graduate up to and including a doctoral degree) for nearly two‐thirds of the sample.

**TABLE 1:** Diagnostic prevalences for mental disorders among university students in Sweden 2020‐23 (*N* = 17,948).

Diagnostic prevalence, % [95% CI]	30 days	12 months	Lifetime
Major depressive disorder	14⋅3 [13⋅6–14⋅9]	23⋅0 [22⋅3–23⋅7]	34⋅1 [33⋅3–34⋅9]
Generalized anxiety disorder	8⋅7 [8⋅3–9⋅2]	15⋅0 [14⋅4–15⋅5]	20⋅8 [20⋅2–21⋅5]
Panic disorder	4⋅3 [3⋅7–4⋅9]	8⋅0 [7⋅2–8⋅9]	10⋅7 [9⋅8–11⋅6]
Bipolar disorder, any	0⋅6 [0⋅4–0⋅8]	1⋅2 [ 0⋅9–1⋅4]	1⋅6 [1⋅3–1⋅9]
Alcohol use disorder	7⋅4 [6⋅6–8⋅2]	15⋅0 [14⋅2–15⋅8]	—
Substance use disorder	0⋅8 [0⋅7–0⋅9]	1⋅8 [1⋅6–2⋅0]	5⋅2 [4⋅9–5⋅6]
Any mental disorder	25⋅2 [24⋅2–26⋅1]	39⋅3 [38⋅3–40⋅3]	44⋅5 [43⋅6–45⋅3]

**TABLE 2:** Diagnostic prevalences for mental disorders among university students in Sweden 2020‐23 (*N* = 17,948), estimated using multilevel logistic regression with Bayesian inference.

Diagnostic prevalence (%)	30 days (posterior median, 95% CoI)^a^	12 months (posterior median, 95% CoI)^a^	Lifetime (posterior median, 95% CoI)^a^
Major depressive disorder	13·9 [12·2; 15·6]	22·7 [19·6; 26·2]	34·2 [31·8; 36·6]
Generalized anxiety disorder	8·8 [7·5; 10·3]	15·1 [12·7; 17·9]	21·3 [19·2; 23·7]
Panic disorder	4·0 [2·2; 7·1]	7·4 [4·3; 12·7]	10·2 [6·3; 16·3]
Bipolar disorder, any	0·6 [0·4; 1·0]	1·2 [0·8; 1·7]	1·7 [1·2; 2·2]
Alcohol use disorder	6·7 [5·0; 8·7]	13·8 [11·4; 16·3]	—
Substance use disorder	0·8 [0·6; 1·0]	1·8 [1·4; 2·1]	5·2 [4·5; 6·0]
Any mental disorder	24·5 [20·7; 28·6]	38·7 [33·6; 44·0]	45.0 [41·2; 48·8]

^a^ Median of the posterior probability distribution of the main intercept of the multilevel regression model converted to proportion (i.e., prevalence) with 95% CoI defined by the 2·5% and 97·5% percentiles of the posterior distributions.

**TABLE 3:** Odds ratios of diagnosis for any mental disorders associated with university student characteristics in Sweden 2020‐23 (*N* = 17,948).

Sociodemographic characteristics	*N*	%	30 days OR [95% CI] *p*	12 months OR [95% CI] *p*	Lifetime OR [95% CI] *p*
Age (*M* = 25·5, SD = 7·9)					
18–19 years	*n* = 2757	15·4%	(ref)	(ref)	(ref)
20–29 years	*n* = 11,353	63·3%	1·26 [1·13–1·41] < 0·0001^a^	1·39 [1·26–1·54] < 0·0001^a^	1·51 [1·37–1·66] < 0·0001^a^
30 years or older	*n* = 3839	21·4%	0·69 [0·60–0·80] < 0·0001^a^	0·70 [0·62–0·80] < 0·0001^a^	1·27 [1·13–1·42] < 0·0001^a^
Gender identity					
Men	*n* = 5133	28·6%	(Ref)	(Ref)	(Ref)
Women	*n* = 12,457	69·4%	1·24 [1·13–1·37] < 0·0001^a^	1·27 [1·17–1·38] < 0·0001^a^	1·76 [1·64–1·90] < 0·0001^a^
Other	*n* = 358	2·0%	1·94 [1·50–2·52] < 0·0001^a^	2·71 [2·11–3·47] < 0·0001^a^	3·38 [2·64–4·32] < 0·0001^a^
Marital status					
Never married or cohabited	*n* = 11,905	66·3%	(Ref)	(Ref)	(Ref)
Married or cohabiting	*n* = 5158	28·7%	0·66 [0·60–0·73] < 0·0001^a^	0·68 [0·63–0·75] < 0·0001^a^	1·04 [0·97–1·13] 0·28
Separated, divorced, or widowed	*n* = 885	4·9%	1·01 [0·84–1·21] 0·94	0·98 [0·83–1·16] 0·79	1·33 [1·13–1·57] 0·0006^a^
Sexual orientation					
Heterosexual: no same‐ sex attraction	*n* = 9299	51·8%	(Ref)	(Ref)	(Ref)
Heterosexual: Some same‐sex attraction	*n* = 3509	19·5%	1·30 [1·17–1·46] < 0·0001^a^	1·46 [1·32–1·61] < 0·0001^a^	1·44 [1·31–1·58] < 0·0001^a^
Nonheterosexual: Without same‐sex Activity	*n* = 3684	20·5%	1·66 [1·49–1·86] < 0·0001^a^	1·89 [1·71–2·09] < 0·0001^a^	2·10 [1·91–2·31] < 0·0001^a^
Nonheterosexual: With same‐sex activity	*n* = 1457	8·1%	1·91 [1·66–2·20] < 0·0001^a^	2·38 [2·06–2·74] < 0·0001^a^	2·59 [2·26–2·97] < 0·0001^a^
Full‐time studies					
Yes	*n* = 16,781	93·5%	(Ref)	(Ref)	(Ref)
No	*n* = 1167	6·5%	0·81 [0·67–0·98] 0·03	0·73 [0·62–0·86] 0·0001^a^	0·96 [0·82–1·12] 0·57
Work in parallel					
No	*n* = 11,773	65·6%	(Ref)	(Ref)	(Ref)
Yes	*n* = 6175		0·87 [0·78–0·96] 0·007	0·90 0·81–0·99] 0·03	0·98 0·90–1·07] 0·63
Hours of study per week					
20 h or less	*n* = 5657	31·5%	1·33 [1·20–1·48] < 0·0001^a^	1·25 [1·14–1·37] < 0·0001^a^	1·12 [1·03–1·22] 0·007
21–40 h	*n* = 9283	51·7%	(Ref)	(Ref)	(Ref)
41 h or more	*n* = 3008	16·8%	0·93 [0·82–1·06] 0·27	0·90 [0·80–1·01] 0·08	1·00 [0·90–1·11] 0·98
University site^b^					
Metropolitan city	*n* = 12,026	67·0%	(Ref)	(Ref)	(Ref)
Non‐metropolitan city	*n* = 5922	33·0%	0·98 [0·89–1·07] 0·64	0·97 [0·87–1·09] 0·65	1·04 [0·93–1·17] 0·45
Respondent's country of birth					
Sweden	*n* = 14,683	81·8%	(Ref)	(Ref)	(Ref)
Other	*n* = 3265	18·2%	0·87 [0·77–0·98] 0·02	0·73 [0·65–0·81] < 0·0001^a^	0·72 [0·66–0·80] < 0·0001^a^
International student					
No	*n* = 16,817	93·7%	(Ref)	(Ref)	(Ref)
Yes^d^	*n* = 1131	6·3%	0·89 [0·75–1·05] < 0·0001^a^	0·83 [0·71–0·96] 0·01	0·73 [0·63–0·84] < 0·0001^a^
Parental country of birth					
Both parents born in Sweden	*n* = 12,065	67·2%	(Ref)	(Ref)	(Ref)
Both parents born in another country	*n* = 3935	21·9%	0·99 [0·88–1·10] 0·80	0·79 [0·71–0·87] < 0·0001^a^	0·83 [0·75–0·91] < 0·0001^a^
One parent born in another country	*n* = 1948	10·9%	1·10 [0·96–1·27] 0·18	0·99 [0·87–1·12] 0·85	1·09 [0·96–1·22] 0·18
Parental education^c^					
High	*n* = 10,639	59·3%	(Ref)	(Ref)	(Ref)
Middle	*n* = 6338	35·3%	1·07 [0·98–1·17] 0·11	1·03 [0·95–1·11] 0·52	1·15 [1·07–1·24] 0·0002^a^
Low	*n* = 971	5·4%	0·99 [0·82–1·18] 0·88	0·81 [0·68–0·96] 0·01	1·03 [0·88–1·20] 0·75
Covid‐19 periods^c^					
Early	*n* = 5552	30·9%	(Ref)	(Ref)	(Ref)
Persisting	*n* = 5329	29·7%	1·08 [0·92–1·26] 0·35	1·05 [0·93–1·18] 0·03	1·13 [1·01–1·26] 0·03
Post‐pandemic	*n* = 7067	39·4%	1·50 [1·31–1·72] < 0·0001^a^	1·54 [1·37–1·73] < 0·0001^a^	1·32 [1·19–1·47] < 0·0001^a^

Abbreviation: OR=Odds ratio.

^a^ Significant *p*‐values following Bonferroni correction at the 0·0008 level.

^b^ Metropolitan city = city with more than 200,000 inhabitants.

^c^ Parental education categories: Low = none, elementary school, or don't know; Middle = secondary school, or some post‐secondary education; High = university graduate, or doctoral degree.

^c^ Early = spring and autumn 2020; Persisting = spring and autumn 2021 and spring 2022; post‐pandemic = autumn 2022 and spring and autumn 2023.

^d^ International students' country of birth and parental country of birth were included in the overall descriptive statistics.

**TABLE 4:** Odds ratios of diagnosis for any mental disorders associated with university student characteristics in Sweden 2020‐23 (*N* = 17,948), estimated using multilevel logistic regression with Bayesian inference.

Sociodemographic characteristics	30 days		12 months		Lifetime	
	Est.	Prob.	Est.	Prob.	Est.	Prob.
Age						
18–19 years	(Ref)		(Ref)		(Ref)	
20–29 years	1·26 (1·13; 1·41)	> 99·9%	1·39 (1·26; 1·54)	> 99·9%	1·51 (1·37; 1·66)	> 99·9%
30 years or older	0·69 (0·60; 0·80)	> 99·9%	0·70 (0·62; 0·80)	> 99·9%	1·27 (1·13; 1·42)	> 99·9%
Gender						
Man	(Ref)		(Ref)		(Ref)	
Woman	1·24 (1·13; 1·37)	> 99·9%	1·27 (1·17; 1·38)	> 99·9%	1·76 (1·64; 1·90)	> 99·9%
Other	1·94 (1·49; 2·51)	> 99·9%	2·70 (2·11; 3·47)	> 99·9%	3·37 (2·65; 4·33)	> 99·9%
Marital status						
Never married or cohabited	(Ref)		(Ref)		(Ref)	
Married or cohabiting	0·66 (0·60; 0·74)	> 99·9%	0·68 (0·63; 0·75)	> 99·9%	1·04 (0·97; 1·13)	86·1%
Separated, divorced, or widowed	1·01 (0·84; 1·20)	52·9%	0·98 (0·83; 1·16)	60·5%	1·33 (1·13; 1·57)	> 99·9%
Sexual orientation						
Heterosexual: no same‐sex attraction	(Ref)		(Ref)		(Ref)	
Heterosexual: Some same‐sex attraction	1·30 (1·17; 1·46)	> 99·9%	1·46 (1·32; 1·61)	> 99·9%	1·44 (1·31; 1·58)	> 99·9%
Nonheterosexual: Without same‐sex activity	1·66 (1·49; 1·86)	> 99·9%	1·89 (1·71; 2·09)	> 99·9%	2·10 (1·92; 2·31)	> 99·9%
Nonheterosexual: With same‐sex activity	1·91 (1·65; 2·19)	> 99·9%	2·38 (2·06; 2·74)	> 99·9%	2·59 (2·27; 2·98)	> 99·9%
Full‐time studies						
Yes	(Ref)		(Ref)		(Ref)	
No	0·82 (0·67; 0·97)	98·8%	0·73 (0·61; 0·85)	> 99·9%	0·96 (0·82; 1·12)	71·5%
Work in parallel						
No	(Ref)		(Ref)		(Ref)	
Yes	0·87 (0·79; 0·96)	99·6%	0·90 (0·82; 0·99)	98·5%	0·98 (0·90; 1·07)	67·9%
Hours of study per week						
20 h or less	1·34 (1·21; 1·48)	> 99·9%	1·25 (1·14; 1·37)	> 99·9%	1·13 (1·03; 1·23)	99·6%
21–40 h	(Ref)		(Ref)		(Ref)	
41 h or more	0·93 (0·82; 1·06)	86·1%	0·90 (0·80; 1·01)	95·8%	1·00 (0·90; 1·11)	50·2%
University site						
Metropolitan city	(Ref)		(Ref)		(Ref)	
Non‐metropolitan city	0·98 (0·87; 1·10)	66·6%	0·97 (0·85; 1·13)	66·7%	1·04 (0·91; 1·20)	74·5%
Respondent's country of birth						
Sweden	(Ref)		(Ref)		(Ref)	
Other	0·87 (0·77; 0·98)	99·1%	0·73 (0·65; 0·81)	> 99·9%	0·72 (0·66; 0·80)	> 99·9%
International student						
No	(Ref)		(Ref)		(Ref)	
Yes	0·89 (0·75; 1·04)	92·6%	0·83 (0·71; 0·96)	99·5%	0·73 (0·63; 0·84)	> 99·9%
Parental country of birth						
Both parents born in Sweden	(Ref)		(Ref)		(Ref)	
Both parents born in another country	0·99 (0·88; 1·11)	60·1%	0·79 (0·71; 0·87)	> 99·9%	0·83 (0·75; 0·91)	> 99·9%
One parent born in another country	1·10 (0·96; 1·27)	91·1%	0·99 (0·87; 1·12)	57·7%	1·09 (0·96; 1·22)	91·2%
Parental education						
High	(Ref)		(Ref)		(Ref)	
Middle	1·08 (0·99; 1·18)	94·7%	1·03 (0·95; 1·11)	73·8%	1·15 (1·07; 1·24)	> 99·9%
Low	0·99 (0·82; 1·18)	56·0%	0·81 (0·69; 0·96)	99·4%	1·03 (0·88; 1·20)	62·4%
Covid‐19 period						
Early	(Ref)		(Ref)		(Ref)	
Persisting	1·05 (0·75; 1·37)	65·3%	1·04 (0·78; 1·35)	64·7%	1·12 (0·88; 1·39)	87·7%
Post‐pandemic	1·49 (1·10; 1·20)	98·9%	1·53 (1·15; 1·20)	99·3%	1·34 (1·08; 1·70)	99·0%

*Note:* Est.: Median of the posterior distribution of the odds ratio associated with the characteristic and 95% CoI defined by the 2·5% and 97·5% percentiles of the posterior distribution. Prob.: The proportion of the posterior distribution in the direction of the mean, that is, the probability of any association.

Diagnostic prevalences for specific disorders and, overall, for any mental disorder, are shown in Tables 1 and 2. Regarding any mental disorder, one out of four respondents fulfilled criteria for any 30d diagnosis, two out of five fulfilled criteria for any 12m diagnosis and almost half fulfilled criteria for any LT diagnosis. Diagnostic prevalences for specific diagnoses were lowest for 30d diagnoses, somewhat higher for 12m diagnoses, and highest for LT diagnoses. The order of prevalences was MDD, GAD and AUD, pertaining to the most common mental disorders, followed by PD, SUD and BD, all less common disorders, at all time periods. An exception was that GAD and AUD shared second place for past 12m diagnoses. We also calculated co‐morbidity by number of diagnoses per participant, finding that it was relatively low for 30d diagnoses, higher for 12m and highest for LT diagnoses. The proportion of the total sample with only one diagnosis (i.e., no comorbidity) ranged between 15% and 25%. Co‐morbidity prevalences ranged between 5% and 15% for two diagnoses across time periods, 2%–5% for three diagnoses, and under 1% for four diagnoses; see Supporting Information S1: Table S2.

Associations between participant characteristics are presented in terms of odds ratios in Tables 3 and 4. Students in their twenties (20–29 years) had higher odds than 18‐19‐year‐olds of reporting data that led to diagnosis of any mental disorder at 30d, 12m, and LT, with odds increasing at each timepoint. Respondents 30 years or older had lower odds than 18–19‐year‐olds of reporting any 30d, 12m disorder, and higher odds of reporting LT mental disorder. Women had higher odds than men of reporting any mental disorder at 30d and 12m, and LT. Respondents with “other” gender identification had even higher odds of reporting any mental disorder at 30d, with markedly higher odds at 12m and LT. Participants who were married or cohabiting had lower odds than those never married of reporting mental disorder at 30d and 12m. In contrast, separated, divorced or widowed respondents had higher odds than those never married of reporting any LT mental disorder.

Respondents identifying as heterosexual with some same‐sex attraction had higher odds of reporting any mental disorder at 30d, 12m and LT compared to heterosexual identifiers without same‐sex attraction (the reference group). Respondents identifying as non‐heterosexual without same‐sex activity had higher odds of reporting any 30d, 12m or LT mental disorder compared to the reference group. In addition, non‐heterosexual identifiers with same‐sex activity had higher odds of reporting any 30d disorder, and markedly higher odds of reporting 12m and LT mental disorder, all in comparison to the reference group.

Respondents with part‐time studies had lower odds than full‐time students of reporting any 12m mental disorder. Respondents who spent 20 h or less per week on their studies had higher odds than those studying 21–40 hours a week of reporting any 30d and 12m mental disorders. Respondents reporting birth outside Sweden had lower odds than respondents born in Sweden of reporting any mental disorder at 12m and LT timepoints. Respondents who identified as international students also had lower odds than domestic students of reporting 30d and LT mental disorders. Respondents whose parents were both born outside Sweden also had lower odds of reporting any 12m or LT mental disorder. Respondents whose parents had a middle level of education had lower odds than those with highly educated parents of reporting LT mental disorders of any kind. Finally, respondents in the post‐pandemic phase had higher odds than respondents early in the pandemic of reporting any mental disorder at the 30d, 12m and LT timepoints.

## 4 Discussion

This study reports mental disorder diagnoses among students in higher education in Sweden based on cross‐sectional mental health data collected systematically in a large‐scale collaborative venture with 15 universities and colleges in Sweden over a four‐year period. Mental health findings in the form of diagnostic prevalences have not previously been reported for students in Sweden and our findings confirm and, indeed, increase existing concern over student mental health in the country.

Over two‐thirds of the respondents in the current study were women, in line with the overrepresentation of women among students in Swedish higher education. The average respondent age was 25, with five out of six older than 20, higher in comparison to other countries (Supporting Information S1: Table S3). The higher age may result from the intentionally flexible and tuition‐free design of public higher education in Sweden, which allows for individually divergent paths of study and accommodates students who enter their studies some years after completing secondary school, also allowing for extended study breaks.

Levels of mental disorders among students in Sweden were high, corresponding to the general picture in a variety of different countries, regardless of high student age and the unique higher education policies in Sweden that allow for some individual study flexibility. We found prevalences of 39⋅3% for 12‐month diagnoses and 44⋅5% for lifetime diagnoses. Prior research showed that corresponding prevalences were only somewhat lower at 31⋅4% and 35⋅3%, respectively, in the multi‐country WMH‐ICS analysis conducted by Auerbach et al. (2018), 31⋅5% and 38⋅5% respectively in South Africa (Bantjes et al. 2019), 34⋅9% for 12‐month diagnoses in Belgium (Bruffaerts et al. 2018), 35⋅7% and 41⋅3% for respective 12‐month and lifetime diagnoses in Spain (Ballester et al. 2020), 38⋅4% and 42⋅2% (for only MDD) in Chile (Crockett et al. 2024), and 47⋅5% and 53⋅2% respectively in Northern Ireland (Mclafferty et al. 2017). In Norway, where the system of higher education with its tuition‐free attendance appears to offer flexibility like that in Sweden, all students in the country were invited to respond to a survey with content similar to the WMH‐ICS questionnaire. Among over 10,000 participants with a mean age similar to Swedish students, reported by gender, past 30‐day prevalences of any mental disorder in Norway were higher than in Sweden, at 39⋅7% among women and 25⋅7% in men, with 57⋅3% and 42⋅5% 12‐month prevalences among women and men, respectively, and 67⋅3% and 53⋅6% lifetime prevalences for women and men, all termed “alarming” by the authors (Sivertsen et al. 2023). In sum, findings in Sweden showed somewhat higher prevalences than in South Africa, Belgium, and Spain, and possibly Chile. Our prevalences were closer to, although slightly lower, than those in Northern Ireland and Norway, possibly suggesting higher mental disorder prevalences in Northern Europe. Supporting Information S1: Table S3 shows a country‐based overview of sample age and gender distributions as well as diagnostic prevalences.

Demographic characteristics associated with lower odds for any recent mental disorder were found for students 30 years or older as compared to 18–19 year olds, while 20–29‐year‐olds had higher odds than the youngest group of students; this finding corresponds approximately with earlier research identifying higher odds of mental disorder in emerging adults in the span of approximately 18–29 years, compared to older adults (Arnett et al. 2014). The gender gap in mental health, consistently showing that women report worse mental health than men, begins in adolescence (Campbell et al. 2021). Our findings align well with this gap given the finding of higher odds for any mental disorder among women at all diagnostic timepoints, suggesting the need to undertake further efforts for minimization of the gender gap.

Turning to possible pandemic effects during our data collection period, we found no evidence for increased odds of mental disorder among individuals in the persisting pandemic phase, compared to the early pandemic period, a finding that appears to correspond to studies that identified a waning effect in mental distress as the pandemic progressed (e.g., Andersson et al. 2024), after initial increases in mental disorder (Berman et al. 2022). We were not able to measure increases in the initial pandemic phase given that our data collection began in the spring of 2020, coinciding with the start of the pandemic. However, separate pandemic research in Sweden among students showed initial negative effects on mental health and academic self‐efficacy, but also a decline during the persisting phase of the pandemic, when voluntary recommendation compliance with restrictions continued to be high (Andersson et al. 2024).

Regarding the post‐pandemic phase, the current study showed consistently higher odds of any mental disorder for all diagnostic timepoints during this period. The lifetime diagnostic association with a higher odds of post‐pandemic mental distress suggests that having a history of some kind of mental disorder may have been associated with a recurrence or persistence phenomenon, where those with past mental distress adjusted to the pandemic conditions in the middle (persisting) phase without harm but increased their distress once societal routines regarding social contact returned to pre‐pandemic conditions. Our finding of higher odds for 30‐day and 12‐month mental disorder in the post‐pandemic phase suggests difficulties in re‐adjusting to a “return to normal”, analogous to findings in a small longitudinal US study comparing findings to 1991 norms (Nails et al. 2023). A significant additional factor that may well have contributed to lower post‐pandemic mental health is the pandemic‐related decline in physical activity among adults 20–35 years old (Wunsch et al. 2022).

We found markedly higher odds for any mental disorder in individuals identifying in some way as LGBTQIA+, including those who gender‐identified as non‐binary as well as for students with non‐heterosexual sexual orientation whether or not they reported same‐sex activity, and for students with heterosexual orientation and same‐sex attraction. It is known that non‐binary gender identity is associated with a higher risk of mental disorder than for cisgender, for example by an effect size of *d =* 0⋅48 in meta‐analysis of individuals 11–25 years old (Klinger et al. 2024). LGBTQ+ sexual orientation is also associated with a higher risk of mental disorder than heterosexual orientation, where a meta‐analysis has shown 32⋅2% lifetime MDD prevalence, compared to 4⋅7% in the global general population (Cai et al. 2024). A WMH‐ICS study from Mexico on students with bisexual identity from a 2016‐2017 cohort showed an odds ratio of 2⋅2 for past 12‐month MDD compared to students with heterosexual identity without same‐sex attraction (Rentería et al. 2021). This aligns with our odds ratio of 2⋅4 for any past 12‐month mental disorder for non‐heterosexual individuals with same‐sex activity, likewise in comparison with heterosexual identifiers without same‐sex attraction. Notably however, our odds ratio concerns *any* mental disorder, an overriding category that includes MDD, so the odds ratio for an MDD diagnosis in our sample would be lower, possibly due to a higher rate of LGBTQ+ acceptance in Sweden (9⋅2) as compared to Mexico (6⋅5) (Flores 2021). Although we did not evaluate concordance of *activity* among heterosexual participants, we do note that the higher odds ratio for mental disorder among heterosexual students in Sweden with *attraction* discordance (i.e., same‐sex attraction) can be a factor signaling mild to moderate mental distress, both recent and lifetime.

### 4.1 Clinical Significance

In this first diagnostic study among students in higher education in Sweden, we found prevalences of mental disorder diagnoses at 25⋅2% for the past 30 days, and considerably higher for the past 12 months at 39⋅3%. Prior large‐scale research on student mental health in Sweden is sparse, but the Swedish Public Health Agency analyzed a national sample of students within the 2014‐2016 National Health Survey, identifying symptom‐based low wellbeing for 25% of students 16–29 years old, compared to 16% of individuals in the same age group who were in the labor market (Swedish Public Health Agency 2018); women were overrepresented among those experiencing low wellbeing. These decade‐old findings concord with our diagnosis‐based findings, although the lower level of diagnoses in symptom‐based data would suggest that the rates of mental disorder have since increased among students. This suggests an urgency for continued measurement of student mental health.

The disorders most diagnosed in our study at all timepoints were MDD and GAD, suggesting that student mental health services (SMHS's) need to be equipped to treat mild to moderate MDD and GAD. AUD, figuring among common disorders for the past 30 days and past 12 months, also needs to be addressed by SMHS's through brief intervention for identified help‐seekers, as well as through consistently maintained prevention measures. The evidence base for effective digital treatment of depression and anxiety, as well as panic disorder is now robust (Hedman‐Lagerlöf et al. 2023) and such low‐threshold interventions should not be withheld from students in the university context. It is less obvious that the less common disorder of BD, requiring more highly specialized treatment provider competence, can and should be addressed within SMHS (Brown 2018; Cage et al. 2021; Priestley et al. 2022); this is particularly relevant in Sweden, where the legislatively dictated focus of SMHS interventions is prevention rather than specialized treatment. Although SUD pertains to the less common disorders in terms of prevalence in our study, brief intervention and prevention efforts should be included in AUD initiatives. Increasing treatment access for students should improve mental health outcomes, increase the probability of degree completion and reduce health‐related inequities, particularly in more vulnerable sub‐groups.

For SMHS's to optimize knowledge about who they reach and systematize counseling outcomes, shared systematic national SMHS data collection would provide a basis for formulating support and intervention strategies adapted to ongoing changes in student needs, as exemplified in a study covering four UK SMHS's (Broglia et al. 2023). The recent publication of WHO guidelines for the implementation of psychological interventions (WHO 2024) indicates the high priority of widening access to and uptake of psychological interventions in order to improve global mental health. This call is highly relevant for the SMHS mandate in Sweden to prevent mental disorder (at primary, secondary levels, and at some HEI's, even at tertiary levels). Expanded access to digital mental health interventions would be a low‐threshold means of approaching fulfillment of this mandate.

### 4.2 Strengths and Limitations

A major strength of this study is that it was the first large scale systematic data collection on common DSM‐5 mental disorder diagnoses among students in Sweden, focusing on the past 30 days, past 12 months and lifetime periods. Limitations included the response rate of 13% to the overall invited cohort (range 9⋅2‐24⋅1% over the eight sub‐cohorts in our sample), a rate similar to the Healthy Minds study, which reported response rates of 13%–27% among US students surveyed 2013‐2021 (Lipson et al. 2022). We argue that the overrepresentation of women corresponds approximately to the pattern of gender‐based recruitment to higher education in Sweden, and that the mean age is typical of students' life trajectory in the young adult years in Sweden. An unanswered question is whether our sample might contain a bias toward respondents with higher or lower levels of mental health, skewing our findings in a pessimistic or optimistic direction. To assess the question of representativity in our imputed data, we conducted a sensitivity analysis comparing diagnostic prevalences in the total imputed sample with those in the partially imputed sample as well as an analysis of non‐imputed data, and found no clear pattern suggesting higher or lower mental health rates in the non‐imputed data compared to the imputed data set (see Supporting Information S1: Table S1). Given the similarity of our diagnostic prevalences with those in other countries, we conclude that student mental health is disordered for a sizable proportion of student bodies, in Sweden and elsewhere, and could be comprehensively addressed through strategic measures to reduce the treatment gap.

### 4.3 Conclusion and a Call to Action

The globally high level of mental disorders among young adults in higher education requires a concerted response to provide effective help that is personally amenable to the diverse individuals who make up student bodies worldwide. University leadership and mental health services in Sweden have excellent prospects for offering low‐threshold primary, secondary as well as tertiary prevention. A perspective focused on promoting flourishing and resilience could appeal more to students and their mentors than a “crisis narrative” (Bantjes et al. 2023), thus dedramatizing the existence of mental health issues that are part of emerging adulthood and that could be eased by appropriate support at the right moment. Secondary prevention programs with self‐tailored menus could be offered to members of subgroups with higher odds of mental disorders. Low‐threshold tailored and personalized tertiary prevention could efficiently reach students who respond to the type of survey used within the WMH‐ICS consortium. Prior WMH‐ICS research has shown that recent MDD is associated with prior suicidal behaviors, prior traumatic experiences, recent dissolution of romantic relationships, challenging arguments with significant others, and students who experienced recent particularly stressful life events (Ebert, Buntrock, et al. 2019), suggesting high value for continuing to utilize this comprehensive survey in the student group.

From a Northern European regional perspective, efforts at all three prevention levels could be delivered at local SMHS's and, for example, be complemented by a specialized national unit for prevention of mental health distress among students, anchored in digital interventions, and resourced to offer human guidance via telephone calls as needed. Recent UK‐based research has indicated that both student and staff prefer an “inclusive and holistic approach…[with] wellbeing…embedded and signposted across the university” (Cage et al. 2021, 1083). For SMHS's to respond to identified needs, several types of support should be available, including *self‐care*, *informal* support via friends or university student organizations, *structured* support via a peer or buddy support system, *study* support as well as *wellbeing* support through counseling and digital mental health treatment and, additionally, smooth liaison with *external specialist healthcare* for less common mental disorders (Brown 2018). Our ongoing randomized controlled trial on tailored and personalized ICBT, targeting depression and anxiety in participants from the 15 universities included in this study (Berman et al. 2024), is one step in this direction.

In sum, all support efforts need to effectively address student distress, including attention to students with functional disabilities. Such new initiatives would naturally include established partnerships with external specialist healthcare providers to ensure optimal and swift response to acute mental distress outside the SMHS remit. Such new prevention and liaison efforts would require additional earmarked resources so SMHS's in Sweden, and elsewhere, could increase their menus for low‐threshold measures at all three prevention tiers, and efficiently secure need‐based referral to external treatment, to promote flourishing and resilience among all students.

## Author Contributions

**Anne H. Berman:** conceptualisation, data curation, formal analysis, funding acquisition, investigation, methodology, project administration, resources, software, writing–original draft, writing–review and editing, **Claes Andersson:** conceptualisation, data curation, funding acquisition, investigation, methodology, project administration, resources, writing–review and editing, **Marcus Bendtsen:** conceptualisation, data curation, formal analysis, investigation, methodology, project administration, resources, software, validiation, writing–review and editing, **Petra Lindfors:** conceptualisation, funding acquisition, investigation, methodology, writing–review and editing, **Nooshin Talebizadeh:** data curation, investigation, resources, writing–review and editing, **Naira Topooco:** conceptualisation, funding acquisition, investigation, software, writing–original draft, writing–review and editing, **Olof Molander:** conceptualisation, data curation, formal analysis, investigation, methodology, software, writing–original draft, writing–review and editing.

## Funding

Authors A. H. B, C. A, NTa, NTo and O. M were funded by Grant No. 2019‐01127 from the Swedish Research Council to author A. H. B. Authors A. H. B and C. A were also funded for separate commissioned analyses by Grant No. 04252‐2021‐2.3.2 from the Swedish Public Health Agency. NTo was also funded for travel grants by The Ryoichi Sasakawa Young Leaders Fellowship Fund (SYLFF) Program for participation in WMH‐ICS yearly conferences, and for a 6‐week research fellowship at the Department of Global Health and Population at Harvard T.H. Chan School of Public Health. PL and MB's contributions to the article were funded by their employers. None of the funders had any role in study design, collection, analysis or interpretation of data; neither did they have any role in the writing of this article, nor in the decision to submit it for publication.

## Ethics Statement

Survey respondents consented to participate in a study on university students' mental health, approved by the National Swedish Ethical Review Authority, ref. nr. 2020‐01465, May 12, 2020, with approved amendments 2020‐02706, June 16, 2020, 2020‐04639, Oct 12, 2020, 2023‐02378‐02, May 6, 2023, 2023‐02393‐02, May 15, 2023, and 2023‐07621‐02, December 27, 2023.

## Consent

All participants were asked to provide their specific consent to survey participation directly following the study information. Once they had provided their consent, they were asked separately whether they agreed to linking of survey data with educational registry outcomes (the Swedish Ladok system). Only participants who provided their consent to survey participation received access to the survey (regardless of consent to registry linkage).

## Conflicts of Interest

Author AHB owns a small private company, Otimus AB, that provides psychotherapy and occasional training and research services. Author MB owns a private company, Alexit AB, that provides digital screening of lifestyle behaviors. Neither of these companies had any influence or involvement in the research reported. All other authors report no conflicts of interest.

## Supporting information


Supporting Information S1

Supporting Information S2

## Data Availability

The data that support the findings of this study are available on request from the corresponding author. The data are not publicly available due to privacy or ethical restrictions.

## References

[mpr70080-bib-0001] Alonso, J. , Z. Liu , S. Evans‐Lacko , et al. 2018. “Treatment Gap for Anxiety Disorders Is Global: Results of the World Mental Health Surveys in 21 Countries.” Depression and Anxiety 35, no. 3: 195–208. 10.1002/da.22711. 29356216 PMC6008788

[mpr70080-bib-0002] American Psychiatric Association . 2013. Diagnostic and Statistical Manual of Mental Disorders. 5th ed. 10.1176/appi.books.9780890425596.

[mpr70080-bib-0003] Andersson, C. , A. H. Berman , P. Lindfors , and M. Bendtsen . 2024. “Effects of COVID‐19 Contagion in Cohabitants and Family Members on Mental Health and Academic Self‐Efficacy Among University Students in Sweden: A Prospective Longitudinal Study.” BMJ Open 14, no. 3: e077396. 10.1136/bmjopen-2023-077396. PMC10936505 38479749

[mpr70080-bib-0004] Arnett, J. J. , R. Žukauskienė , and K. Sugimura . 2014. “The New Life Stage of Emerging Adulthood at Ages 18–29 Years: Implications for Mental Health.” Lancet Psychiatry 1, no. 7: 569–576. 10.1016/S2215-0366(14)00080-7. 26361316

[mpr70080-bib-0005] Auerbach, R. P. , P. Mortier , R. Bruffaerts , et al. 2018. “WHO World Mental Health Surveys International College Student Project: Prevalence and Distribution of Mental Disorders.” Journal of Abnormal Psychology 127, no. 7: 623–638. 10.1037/abn0000362. 30211576 PMC6193834

[mpr70080-bib-0006] Babor, T. J. , J. C. Higgins‐Biddle , J. B. Saunders , and M. G. Monteiro . 2001. AUDIT. The Alcohol Use Disorders Test. Guidelines for Use in Primary Care. World Health Organization. Department of Mental Health and Substance Dependence.

[mpr70080-bib-0007] Ballester, L. , I. Alayo , G. Vilagut , et al. 2020. “Mental Disorders in Spanish University Students: Prevalence, Age‐of‐Onset, Severe Role Impairment and Mental Health Treatment.” Journal of Affective Disorders 273: 604–613. 10.1016/j.jad.2020.04.050. 32560960

[mpr70080-bib-0008] Bantjes, J. , X. Hunt , and D. J. Stein . 2023. “Anxious, Depressed, and Suicidal: Crisis Narratives in University Student Mental Health and the Need for a Balanced Approach to Student Wellness.” International Journal of Environmental Research and Public Health 20, no. 6: 4859. 10.3390/ijerph20064859. 36981766 PMC10049682

[mpr70080-bib-0009] Bantjes, J. , C. Lochner , W. Saal , et al. 2019. “Prevalence and Sociodemographic Correlates of Common Mental Disorders Among First‐Year University Students in Post‐Apartheid South Africa: Implications for a Public Mental Health Approach to Student Wellness.” BMC Public Health 19, no. 1: 922. 10.1186/s12889-019-7218-y. 31291925 PMC6617861

[mpr70080-bib-0010] Berman, A. H. , M. Bendtsen , O. Molander , et al. 2022. “Compliance With Recommendations Limiting COVID‐19 Contagion Among University Students in Sweden: Associations With Self‐Reported Symptoms, Mental Health and Academic Self‐Efficacy.” Scandinavian Journal of Public Health 50, no. 1: 70–84. 10.1177/14034948211027824. 34213359 PMC8808007

[mpr70080-bib-0011] Berman, A. H. , N. Topooco , P. Lindfors , et al. 2024. “Transdiagnostic and Tailored Internet Intervention to Improve Mental Health Among University Students: Research Protocol for a Randomized Controlled Trial.” Trials 25, no. 1: 158. 10.1186/s13063-024-07986-1. 38429834 PMC10908025

[mpr70080-bib-0012] Blanco, C. , M. Okuda , C. Wright , et al. 2008. “Mental Health of College Students and Their Non–College‐Attending Peers.” Archives of General Psychiatry 65, no. 12: 1429. 10.1001/archpsyc.65.12.1429. 19047530 PMC2734947

[mpr70080-bib-0013] Broglia, E. , G. Ryan , C. Williams , et al. 2023. “Profiling Student Mental Health and Counselling Effectiveness: Lessons From Four UK Services Using Complete Data and Different Outcome Measures.” British Journal of Guidance and Counselling 51, no. 2: 204–222. 10.1080/03069885.2020.1860191.

[mpr70080-bib-0014] Brown, J. S. L. 2018. “Student Mental Health: Some Answers and More Questions.” Journal of Mental Health 27, no. 3: 193–196. 10.1080/09638237.2018.1470319. 29768071

[mpr70080-bib-0015] Bruffaerts, R. , P. Mortier , G. Kiekens , et al. 2018. “Mental Health Problems in College Freshmen: Prevalence and Academic Functioning.” Journal of Affective Disorders 225: 97–103. 10.1016/j.jad.2017.07.044. 28802728 PMC5846318

[mpr70080-bib-0016] Cadigan, J. M. , C. M. Lee , and M. E. Larimer . 2019. “Young Adult Mental Health: A Prospective Examination of Service Utilization, Perceived Unmet Service Needs, Attitudes, and Barriers to Service Use.” Prevention Science 20, no. 3: 366–376. 10.1007/s11121-018-0875-8. 29411197 PMC6081266

[mpr70080-bib-0017] Cage, E. , E. Jones , G. Ryan , G. Hughes , and L. Spanner . 2021. “Student Mental Health and Transitions Into, Through and Out of University: Student and Staff Perspectives.” Journal of Further and Higher Education 45, no. 8: 1076–1089. 10.1080/0309877x.2021.1875203.

[mpr70080-bib-0018] Cai, H. , P. Chen , Q. Zhang , et al. 2024. “Global Prevalence of Major Depressive Disorder in LGBTQ+ Samples: A Systematic Review and Meta‐Analysis of Epidemiological Studies.” Journal of Affective Disorders 360: 249–258. 10.1016/j.jad.2024.05.115. 38795782

[mpr70080-bib-0019] Campbell, O. L. K. , D. Bann , and P. Patalay . 2021. “The Gender Gap in Adolescent Mental Health: A Cross‐National Investigation of 566,829 Adolescents Across 73 Countries.” SSM, Population Health 13: 100742. 10.1016/j.ssmph.2021.100742. 33748389 PMC7960541

[mpr70080-bib-0020] Crockett, M. A. , V. Martínez‐Nahuel , S. Mac‐Ginty , D. Núñez , Á. I. Langer , and J. Gaete . 2024. “Differences in Mental Health Problems in LGBT+ First Year College Students in Chile During the Pandemic.” Social Psychiatry and Psychiatric Epidemiology 59, no. 12: 2339–2349. 10.1007/s00127-024-02683-5. 38819521 PMC11522124

[mpr70080-bib-0021] Cuijpers, P. , R. P. Auerbach , C. Benjet , et al. 2019. “The World Health Organization World Mental Health International College Student Initiative: An Overview.” International Journal of Methods in Psychiatric Research 28, no. 2: e1761. 10.1002/mpr.1761. 30614123 PMC6590455

[mpr70080-bib-0022] Ebert, D. D. , C. Buntrock , P. Mortier , et al. 2019. “Prediction of Major Depressive Disorder Onset in College Students.” Depression and Anxiety 36, no. 4: 294–304. 10.1002/da.22867. 30521136 PMC6519292

[mpr70080-bib-0023] Ebert, D. D. , P. Mortier , F. Kaehlke , et al. 2019. “Barriers of Mental Health Treatment Utilization Among First‐Year College Students: First Cross‐National Results From the WHO World Mental Health International College Student Initiative.” International Journal of Methods in Psychiatric Research 28, no. 2: e1782. 10.1002/mpr.1782. 31069905 PMC6522323

[mpr70080-bib-0024] Flores, A. R. 2021. “Social Acceptance of LGBTI People in 175 Countries and Locations: 1981 to 2020.” https://williamsinstitute.law.ucla.edu/wp‐content/uploads/Global‐Acceptance‐Index‐LGBTI‐Nov‐2021.pdf.

[mpr70080-bib-0025] Hedman‐Lagerlöf, E. , P. Carlbring , F. Svärdman , H. Riper , P. Cuijpers , and G. Andersson . 2023. “Therapist‐Supported Internet‐Based Cognitive Behaviour Therapy Yields Similar Effects as Face‐to‐Face Therapy for Psychiatric and Somatic Disorders: An Updated Systematic Review and Meta‐Analysis.” World Psychiatry 22, no. 2: 305–314. 10.1002/wps.21088. 37159350 PMC10168168

[mpr70080-bib-0026] Kazdin, A. E. 2017. “Addressing the Treatment Gap: A Key Challenge for Extending Evidence‐Based Psychosocial Interventions.” Behaviour Research and Therapy 88: 7–18. 10.1016/j.brat.2016.06.004. 28110678

[mpr70080-bib-0027] Kessler, R. C. , and T. B. Üstün . 2004. “The World Mental Health (WMH) Survey Initiative Version of the World Health Organization (WHO) Composite International Diagnostic Interview (CIDI).” International Journal of Methods in Psychiatric Research 13, no. 2: 93–121. 10.1002/mpr.168. 15297906 PMC6878592

[mpr70080-bib-0028] Klinger, D. , S.‐M. Oehlke , S. Riedl , et al. 2024. “Mental Health of Non‐binary Youth: A Systematic Review and Meta‐Analysis.” Child and Adolescent Psychiatry and Mental Health 18, no. 1: 126. 10.1186/s13034-024-00822-z. 39385290 PMC11465615

[mpr70080-bib-0029] Lederer, A. M. , and M. T. Hoban . 2022. “The Development of the American College Health Association‐National College Health Assessment III: An Improved Tool to Assess and Enhance the Health and Well‐Being of College Students.” Journal of American College Health 70, no. 6: 1606–1610. 10.1080/07448481.2020.1834401. 33400616

[mpr70080-bib-0030] Lipson, S. K. , S. Zhou , S. Abelson , et al. 2022. “Trends in College Student Mental Health and Help‐Seeking by Race/Ethnicity: Findings From the National Healthy Minds Study, 2013–2021.” Journal of Affective Disorders 306: 138–147. 10.1016/j.jad.2022.03.038. 35307411 PMC8995361

[mpr70080-bib-0031] Mclafferty, M. , C. R. Lapsley , E. Ennis , et al. 2017. “Mental Health, Behavioural Problems and Treatment Seeking Among Students Commencing University in Northern Ireland.” PLoS One 12, no. 12: e0188785. 10.1371/journal.pone.0188785. 29236727 PMC5728481

[mpr70080-bib-0032] Nails, J. G. , J. Maffly‐Kipp , H. L. DeShong , S. E. Lowmaster , and J. E. Kurtz . 2023. “A Crisis in College Student Mental Health? Self‐Ratings of Psychopathology Before and After the COVID‐19 Pandemic.” Psychological Assessment 35, no. 11: 1010–1018. 10.1037/pas0001241. 37289503

[mpr70080-bib-0033] Priestley, M. , E. Broglia , G. Hughes , and L. Spanner . 2022. “Student Perspectives on Improving Mental Health Support Services at University.” Counselling and Psychotherapy Research 22, no. 1: capr.12391. 10.1002/capr.12391.

[mpr70080-bib-0034] Rentería, R. , C. Benjet , R. A. Gutiérrez‐García , et al. 2021. “Prevalence of 12‐month Mental and Substance Use Disorders in Sexual Minority College Students in Mexico.” Social Psychiatry and Psychiatric Epidemiology 56, no. 2: 247–257. 10.1007/s00127-020-01943-4. 32886133

[mpr70080-bib-0035] Santomauro, D. F. , A. M. Mantilla Herrera , J. Shadid , et al. 2021. “Global Prevalence and Burden of Depressive and Anxiety Disorders in 204 Countries and Territories in 2020 Due to the COVID‐19 Pandemic.” Lancet 398, no. 10312: 1700–1712. 10.1016/S0140-6736(21)02143-7. 34634250 PMC8500697

[mpr70080-bib-0036] Sivertsen, B. , A. K. S. Knudsen , B. Kirkøen , et al. 2023. “Prevalence of Mental Disorders Among Norwegian College and University Students: A Population‐based cross‐sectional Analysis.” Lancet Regional Health—Europe 34: 100732. 10.1016/j.lanepe.2023.100732. 37927428 PMC10624983

[mpr70080-bib-0037] Swedish Public Health Agency . 2018. “Psykisk ohälsa bland högskole‐och universitetsstudenter kan förebyggas [Mental Health Issues Among College and University Students can be Prevented].” https://www.folkhalsomyndigheten.se/publikationer‐och‐material/publikationsarkiv/p/psykisk‐ohalsa‐bland‐hogskole‐och‐universitetsstudenter‐kan‐forebyggas/?pub=53659.

[mpr70080-bib-0038] WHO . 2022. “World Mental Health Report: Transforming Mental Health for all.” https://www.who.int/publications/i/item/9789240049338.

[mpr70080-bib-0039] WHO . 2024. “Psychological Interventions Implementation Manual: Integrating Evidence‐Based Psychological Interventions Into Existing Services.” https://www.who.int/publications/i/item/9789240087149.

[mpr70080-bib-0040] Wunsch, K. , K. Kienberger , and C. Niessner . 2022. “Changes in Physical Activity Patterns Due to the Covid‐19 Pandemic: A Systematic Review and Meta‐Analysis.” International Journal of Environmental Research and Public Health 19, no. 4: 2250. 10.3390/ijerph19042250. 35206434 PMC8871718

