# Supplementary material for: High Prevalences of Current Mental Disorder Diagnoses Among University Students in Sweden: A Cross‐Sectional Study With Implications for Student Mental Health Services
Source: Int J Methods Psychiatr Res. 2026 May 27;35(2):e70080. doi: 10.1002/mpr.70080 (PMC13240059; doi:10.1002/mpr.70080)
Supplement: Supplementary file 1 — Supporting Information S1 [file MPR-35-e70080-s001.docx]

**Supplementary Table 1. Sensitivity analysis assessing sample representativity via diagnostic prevalences for the total imputed, partially imputed and original non-imputed samples.**

| **Diagnostic**  **prevalence** | **Imputed samples, % (range across 200 imputed datasets)** | | **Original**  **non-imputed data**  **(N=17 948)** |
| --- | --- | --- | --- |
|  | **Total imputed sample^a^**  **(N=17 948)** | **Partially imputed sample^b^**  **(n=13 173)** |  |
| Major Depressive Disorder |  |  | % (complete n  diagnostic outcomes) |
| 30 days | 14·3 (​​13·8-14·8) | 14·3 (13·8-14·8) | 12·5 (n=13 238) |
| 12 months | 23·0 (22·6-23·5) | 23·7 (23·4-24·2) | 20·6 (n=13 206) |
| Lifetime | 34·1 (33·6-34·8) | 35·5 (35·1-36·1) | 33·0 (n=13 018) |
| Generalized anxiety disorder |  |  |  |
| 30 days | 8·7 (8·6-9·0) | 8·9 (8·8-9·0) | 8·2 (n=16 684) |
| 12 months | 15·0 (14·8-15·3) | 15·6 (15·5-15·7) | 14·0 (n=16 658) |
| Lifetime | 20·8 (20·6-21·2) | 21·9 (21·8-21·9) | 21·6 (n=15 896) |
| Panic Disorder |  |  |  |
| 30 days | 4·3 (3·6-5·0) | 4·5 (3·7-5·2) | 2·3 (n=9 174) |
| 12 months | 8·0 (7·2-9·1) | 8·6 (7·6-9·9) | 3·9 (n=8 931) |
| Lifetime | 10·7 (9·7-12·0) | 11·4 (10·5-12·7) | 6·8 (n=8 739) |
| Bipolar Disorder, any |  |  |  |
| 30 days | 0·6 (0·5-0·8) | 0·7 (0·6-1·0) | 0·5 (n=12 475) |
| 12 months | 1·2 (0·9-1·5) | 1·4 (1·1-1·7) | 0·9 (n=12 471) |
| Lifetime | 1·6 (1·3-2·0) | 1·9 (1·5-2·3) | 1·3 (n=12 476) |
| Alcohol Use Disorder |  |  |  |
| 30 days | 7·4 (6·4-8·5) | 9·5 (8·2-10·9) | 4·7 (n=11 329) |
| 12 months | 15·0 (14·3-16·0) | 19·3 (18·3-20·5) | 12·1 (n=11 658) |
| Substance Use Disorder |  |  |  |
| 30 days | 0·8 (0·8-0·8) | 1·0 (1·0-1·0) | 0·8 (n=17 948) |
| 12 months | 1·8 (1·8-1·8) | 2·3 (2·3-2·3) | 1·8 (n=17 948) |
| Lifetime | 5·2 (5·2-5·2) | 6·6 (6·6-6·6) | 5·2 (n=17 948) |

Note:

a. Completed at least section B of the survey, providing enough data for imputation of criteria included in diagnostic algorithms.

b. Completed section K of the survey, providing non-imputed criteria included in diagnostic algorithms.

**Supplementary Table 2. Number of mental disorder diagnoses per individual in the total sample** **(N=17 948)**

| **n (%)** | **No**  **diagnosis** | **One**  **diagnosis** | **Two**  **diagnoses** | **Three diagnoses** | **Four**  **diagnoses** | **Five**  **diagnoses** | **Six**  **diagnoses** | **Mean number of diagnoses (SD)** |
| --- | --- | --- | --- | --- | --- | --- | --- | --- |
| 30 days | 13 432 (74·8%) | 2 974 (16·6%) | 1 165 (6·5%) | 338 (1·9%) | 36 (0·2%) | <5 (0·0%) | 0 (0·0%) | 0·36 (0·71) |
| 12 months | 10 896 (60·7%) | 3 868 (21·5%) | 2 121 (11·8%) | 877 (4·9%) | 167 (0·9%) | 18 (0·1%) | <5 (0·0%) | 0·64 (0·94) |
| Lifetime | 9970 (55·5%) | 4152 (23·1%) | 2745 (15·3%) | 958 (5·3%) | 120 (0·7%) | <5 (0·0%) | 0 (0·0%) | 0·72 (0·95) |

Note: The diagnoses included were: Major Depression, Generalized anxiety disorder, Panic Disorder, Bipolar Disorder (any), Alcohol Use Disorder and Substance Use Disorder.

**Supplementary Table 3. Research overview of Any mental disorder diagnosis prevalences per country and timepoint.**

| **Author, year** | **Country**  **sample size** | **Mean age/ % over 20; women/men/other (%)** | **30 days**  **(%)** | **12 months**  **(%)** | **Lifetime**  **(%)** |
| --- | --- | --- | --- | --- | --- |
| Berman et al 2024 | Sweden  N=17 948 | 25·5/84·7% (>20);  69·4/28·6/2·0 | 25·2 | 39·3 | 44·5 |
| Auerbach et al 2018 | Multi-country  N=13 084 | - /22·1% (>20);  54·4/45·6/- | - | 31·4 | 35·3 |
| Ballester et al 2020 | Spain  N=2118 | 18·8/-;  55·4/44·6/- | - | 35·7 | 41·3 |
| Bantjes et al 2019 | South Africa  N=1402 | -/7·7 (>21);  55·2/44·8/- | - | 31·5 | 38·5 |
| Bruffaerts et al 2018 | Belgium  N=4921 | -/26·1 (> 19);  55·5/44·6 | - | 34·9 | n/a |
| Crockett et al 2024 | Chile  N=7213 | -/22·3 (>20);  53·2/43·6/3·3 | - | 38·4 (MDD only) | 42·2 (MDD only) |
| McLafferty et al 2017 | N. Ireland  N=739 | 21/-;  62·5/37/.04 | - | 47·5 | 53·2 |
| Sivertsen et al 2023 (SHOT) | Norway  N=10460 | 24/53·7 (> 23);  66·4/33·6/- | 39·7W/25·7M | 57·3W/42·5M | 67·3W/53·6M |
| Lipson et al 2022 (Healthy Minds) | USA  N= 359 777 | -/39·9 (> 22);  56·8/40·5/2·7 | 63·1 (any symptoms) |  |  |
| Lederer & Hoban 2022 (ACHA-NCHA)  <https://www.acha.org/ncha/> | USA 2000-2019  N>2 000 000 | Yearly reports produced. |  |  |  |

**Supplementary Material 1**

**Multiple imputation procedure**

Missing data were imputed using multiple imputation with chained equations, generating 200 datasets over 30 iterations (Enders et al., 2016). The survey included four types of missing data:

1. Missing values related to mental health impairment, required for the DSM-5 diagnostic algorithm but only included in version 3·2 of the survey. This data was unavailable for cohorts 1-4, which used earlier survey versions.
2. Missing values resulting from diagnostic randomization groups, employed to reduce survey length for some respondents.
3. Missing values due to the voluntary skipping of survey items, as most items were not mandatory.
4. Missing values caused by skip programming within the survey design.

The imputation process followed a stepwise approach.

**Step 1: Imputation of the DSM-5 Impair_12m Variable**

In the first step, the DSM-5 variable *Impair_12m* from survey version 3·2 was imputed across all survey versions. Initially, *Impair_12m* was estimated based on the *MentImp12m_1* and *MentImp12m_2* variables, as specified in the DSM-5 version 3·2 algorithm. Missing data for *Impair_12m* was then imputed including the following variables in the imputation:

- *Impair_12m*
- *MentHProb_1*, *MentHProb_2*, *MentHProb_3*
- *EmoProbEver_1*, *EmoProbEver_4*
- *PhysMent_Inter*
- *SocLif_FreqGrp*, *SocLif_FreqHang*
- *MentImp12m_1*, *MentImp12m_2*

The imputed *Impair_12m* variable was added to the original survey dataset by calculating the median value across the 200 imputed datasets.

**Step 2: Application of the DSM-5 Algorithm and Imputation of Diagnostic Outcomes**

In the second step, the R-based DSM-5 version 3·2 diagnostic algorithm was applied to the original survey dataset, which included versions 3·0 (*n* = 5,552), 3·1 (*n* = 4,536), and 3·2 (*n* = 7,860). This enabled the generation of DSM-5 diagnostic outcomes across all survey versions, which included missing data (see above).

Subsequently, a second round of multiple imputation was performed. This included the diagnostic outcomes as well as additional variables relevant to the planned analyses. The imputation included the following variables:

- Diagnostic outcomes: *LT_MDD*, *MDD_12M*, *MDD_30D*, *LT_GAD*, *GAD_12M*, *GAD_30D*, *LT_PD*, *PD_12M*, *PD_30D*, *LT_BP_A*, *BP_A_12M*, *BP_A_30D*, *AUD_12M*, *Alc_prob_dep_30D*, *LT_SUD*, *SUD_12M*, *SUD_30D*
- Survey details: *Survey_BaselineVersion*, *Cohort_code_Fas1*, *Sun_code_Level1*
- Demographics: *Age*, *Gender*, *GenderBirth*, *GenderIdent*, *SexOrient*, *SexAttr_Women*, *SexAttr_Men*, *SexPartGender*, *MaritalStatus*, *RelatStatus*
- Educational and employment variables: *Study_fulltime*, *Hours_stud_week*, *Work_parallel*, *University_name*
- Background characteristics: *Resp_born_Swe*, *InterStud*, *Parents_born_Swe*, *Parent_Educ*
- Health variables: *PhysH12m_Rate*, *MentH12m_Rate*, *HlthLim_1*, *HlthLim_2*, *MentImp12m_1*, *MentImp12m_2*, *PhysHProb_1*, *PhysHProb_2*, *PhysHProb_3*, *PhysHProb_4*, *MentHProb_1*, *MentHProb_2*, *MentHProb_3*, *PhysMent_Inter*
- Recent health indicators: *Hlth30d_1*, *Hlth30d_2*, *Hlth30d_3*, *Hlth30d_4*, *Hlth30d_5*, *Hlth30d_6*, *Pain_Inter*
- Emotional problems: *EmoProbEver_1*, *EmoProbEver_2*, *EmoProbEver_3*, *EmoProbEver_4*, *EmoProbEver_5*
- Survey completion progress: *Progress_section_B_non_completers*, *Progress_section_K_completers*

**Step 3: Analyses**

The third step included the formal analyses in the study (see Statistical analysis in the manuscript and the pre-registered analysis plan). The analyses were conducted on pooled data, across the 200 imputed diagnostic datasets obtained in Step 2, according to Rubin’s rules (Rubin, 1987).

**References**

[Enders, C. K., Mistler, S. A., & Keller, B. T. (2016). Multilevel multiple imputation: A review and evaluation of joint modeling and chained equations imputation. *Psychological Methods*, *21*(2), 222.](https://www.zotero.org/google-docs/?zH8QVG)

Rubin, D.B. (1987) Multiple Imputation for Nonresponse in Surveys. John Wiley & Sons Inc., New York.http://dx.doi.org/10·1002/9780470316696
